# Impact of a New Gynecologic Oncology Hashtag During Virtual-Only ASCO Annual Meetings: An X (Twitter) Social Network Analysis

**DOI:** 10.2196/45291

**Published:** 2024-08-14

**Authors:** Geetu Bhandoria, Esra Bilir, Christina Uwins, Josep Vidal-Alaball, Aïna Fuster-Casanovas, Wasim Ahmed

**Affiliations:** 1 Department of Obstetrics & Gynaecology, Command Hospital Kolkata, Kolkata, India; 2 Department of Global Health, Koç University Graduate School of Health Sciences, Istanbul, Turkey; 3 Department of Obstetrics and Gynecology, University Hospitals Schleswig-Holstein, Kiel, Germany; 4 Royal Surrey NHS Foundation Trust, Surrey, United Kingdom; 5 Health Promotion in Rural Areas Research Group, Gerència d'Atenció Primària i a la Comunitat de la Catalunya Central, Institut Català de la Salut, Manresa, Spain; 6 Faculty of Medicine, University of Vic-Central University of Catalonia, Vic, Spain; 7 Unitat de Suport a la Recerca de la Catalunya Central, Fundació Institut Universitari per a la Recerca a l'Atenció Primària de Salut Jordi Gol i Gurina, Manresa, Spain; 8 eHealth Lab Research Group, School of Health Sciences and eHealth Centre, Universitat Oberta de Catalunya (UOC), Barcelona, Spain; 9 Stirling Management School, Stirling University, FK9 4LA, Stirling, United Kingdom, 44 1482 466914

**Keywords:** social media, academic tweeting, hashtag, gynecologic oncology, Twitter, ASCO, gynecology, oncology, virtual, engagement, software application, users, cancer, social network, health promotion

## Abstract

**Background:**

Official conference hashtags are commonly used to promote tweeting and social media engagement. The reach and impact of introducing a new hashtag during an oncology conference have yet to be studied. The American Society of Clinical Oncology (ASCO) conducts an annual global meeting, which was entirely virtual due to the COVID-19 pandemic in 2020 and 2021.

**Objective:**

This study aimed to assess the reach and impact (in the form of vertices and edges generated) and X (formerly Twitter) activity of the new hashtags #goASCO20 and #goASCO21 in the ASCO 2020 and 2021 virtual conferences.

**Methods:**

New hashtags (#goASCO20 and #goASCO21) were created for the ASCO virtual conferences in 2020 and 2021 to help focus gynecologic oncology discussion at the ASCO meetings. Data were retrieved using these hashtags (#goASCO20 for 2020 and #goASCO21 for 2021). A social network analysis was performed using the NodeXL software application.

**Results:**

The hashtags #goASCO20 and #goASCO21 had similar impacts on the social network. Analysis of the reach and impact of the individual hashtags found #goASCO20 to have 150 vertices and 2519 total edges and #goASCO20 to have 174 vertices and 2062 total edges. Mentions and tweets between 2020 and 2021 were also similar. The circles representing different users were spatially arranged in a more balanced way in 2021. Tweets using the #goASCO21 hashtag received significantly more responses than tweets using #goASCO20 (75 times in 2020 vs 360 times in 2021; *z* value=16.63 and *P*<.001). This indicates increased engagement in the subsequent year.

**Conclusions:**

Introducing a gynecologic oncology specialty–specific hashtag (#goASCO20 and #goASCO21) that is related but different from the official conference hashtag (#ASCO20 and #ASCO21) helped facilitate discussion on topics of interest to gynecologic oncologists during a virtual pan-oncology meeting. This impact was visible in the social network analysis.

## Introduction

X (formerly Twitter) has emerged as one of the social media platforms most frequently used by health care professionals [1]. In addition to individuals sharing information and networking, several academic groups, scientific societies, medical journals, and conference organizers use Twitter for educational purposes [2-4]. The reach and impact of conference hashtags have been studied previously [5-7]. Scientific conferences and academic meetings promote dedicated “conference hashtags” and encourage attendees to share their insights, experiences, and learning on the web through social media. Similarly, a study demonstrated the significant impact of a social media ambassador program during the European Society of Gynaecological Oncology (ESGO) congresses on Twitter, highlighting substantial increases in engagement metrics and follower growth, thus advocating for the efficacy of such initiatives in enhancing congress-related engagement and visibility [8]. Furthermore, another study assessed the impact and reach of the 2020 World Gynecologic Oncology Day Twitter campaign, revealing significant participation from health care professionals and the effectiveness of the #WorldGODay hashtag in raising awareness for gynecologic cancers [9].

The official hashtag is announced in advance and widely disseminated on various social media channels [3]. These hashtags are also displayed across conference venues, and some conferences even display live tweeting during designated scientific sessions or plenaries. The aim is to disseminate meeting information and learning to attendees as well as the wider scientific community.

The COVID-19 pandemic has had a profound impact on scientific conferences. Many meetings were canceled, and others became virtual. Going virtual has affected the use of Twitter during meetings. Beste et al [10] found that the number of tweets and Twitter users at a virtual conference compared to the previous year’s in-person meeting reflected the decline in the number of registrations between the 2 years.

The American Society of Clinical Oncology (ASCO) annual meeting has used its official hashtag, #ASCO, since 2011 [11]. ASCO meetings are one of the largest gatherings of oncology professionals globally. Conversations on the web and offline center around particular topics of interest, subspecialties, and the latest evidence. The COVID-19 pandemic forced both the 2020 and 2021 ASCO meetings to be held virtually. New hashtags (#goASCO20 and #goASCO21) were created for the ASCO virtual conferences in 2020 and 2021 to encourage focused gynecologic oncology discussions at the ASCO meetings. As ASCO meetings cover all oncology topics, subspecialties conversations relating to particular tumor types or subspecialties could get lost in the general discussion. Our study aimed to investigate the impact of virtualization on Twitter engagement during virtual-only ASCO annual meetings, with a focus on gynecologic oncology, and explore strategies for enhancing focused discussions and knowledge dissemination through dedicated conference hashtags.

## Methods

### Data Collection

Twitter data were retrieved using the hashtags #goASCO20 and #goASCO21 for 2020 and 2021, respectively. Data from the whole year were retrieved from the year each conference took place (from January to December) for each meeting using the Academic Track Twitter application programming interface, which provides access to all tweets [12].

### Data Analysis

Data (influential users, topics, web sources, and social network analysis) were analyzed using social network analysis in the NodeXL software application (Social Media Research Foundation) [10], allowing an understanding of the shape of the conversation. Both graphs’ vertices were clustered using the Clauset-Newman-Moore cluster algorithm to generate network visuals. The graphs were then laid out using the Harel-Koren Fast Multiscale layout algorithm. Authors in previous publications have used this methodology successfully [13-15]. Circles with lines between them represent individual Twitter users or accounts: the “mentions” and “replies.” The size of the circles means how influential the user is, with bigger circles representing more influential users. The visuals presented illustrate the interactions between Twitter users. Multimedia Appendix 1provides a compiled list of terms related to social media research for readers’ ease of understanding. We also applied a 2-proportion *z* test to determine whether the change in response rates between 2020 and 2021 were statistically significant. This allowed an understanding of the shape of connections resulting from conversations to be visualized.

Visuals were created to provide an overview of the resulting social networks. Dots represent users. The green lines shown between users are known as “edges.” Edges indicate both the presence and strength of a relationship between a user. There is an edge for each “reply” and “mention” and a “self-loop edge” for each tweet that is neither a “reply” nor a “mention.” The “betweenness centrality” score was used to rank the size of the nodes. This score measures the influence of an individual “vertex” (an individual Twitter user, also referred to as a “node”) on the flow of information between all other “vertices.” This score assumes that information flows along the shortest paths between vertices. In each group, various color dots are bigger than others, indicating that these users are more influential. In addition, green lines from these groups indicate a serious relationship with other users and highlight how they have a strong influence.

### Ethical Considerations

This study gained ethical approval from Newcastle University (Ref: 26055/2022). Twitter users who have been named in the study were personally contacted by the authors and provided their consent before their names or Twitter handles were published.

## Results

### Overview of the Social Networks

**Figure 1.**
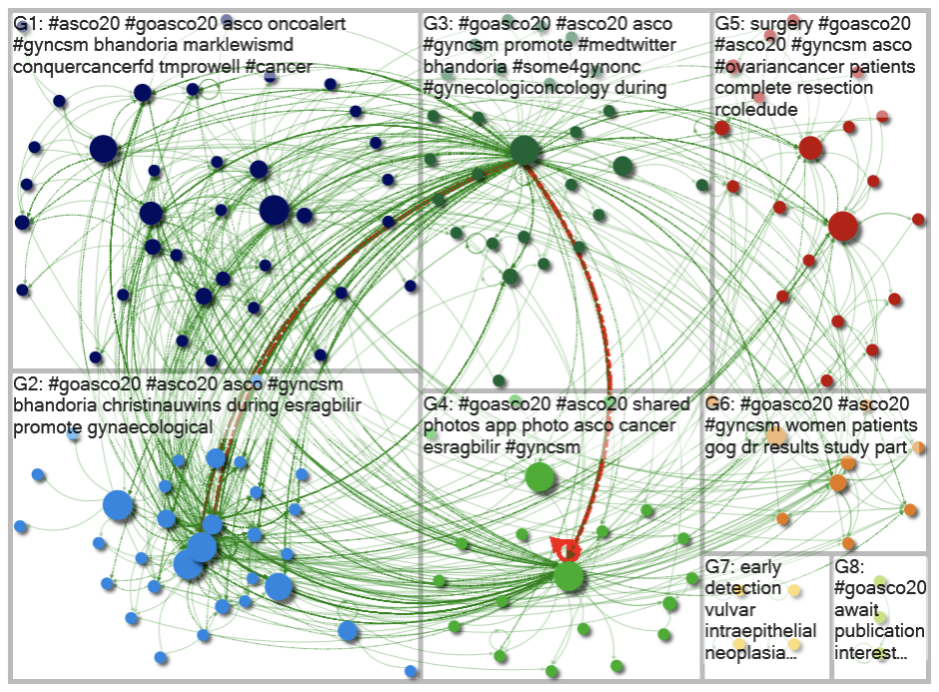
A visual overview of the #goASCO20 Twitter network. ASCO: American Society of Clinical Oncology; G: group.

The most frequently used words or hashtags are highlighted in each group in Figure 1. At the top right of each group, the most used hashtags in order of interaction can be seen. For example, in group 1, the hashtag used the most was #ASCO20, while in groups 2, 3, and 4, it was #goASCO20. It is evident from the figure that different groups discussed varied topics, as depicted by other hashtags apart from #ASCO20 and #goASCO20. Figure 1 illustrates how the various communities of users shared and tweeted the #goASCO20 hashtag. Groups 1, 2, 3 and 4 have an increased number of green lines between them, indicating that their users were tweeting and mentioning one another frequently. Group 3 additionally has red lines connecting itself to groups 2 and 4. The red lines indicate stronger connections in social networks.

**Figure 2.**
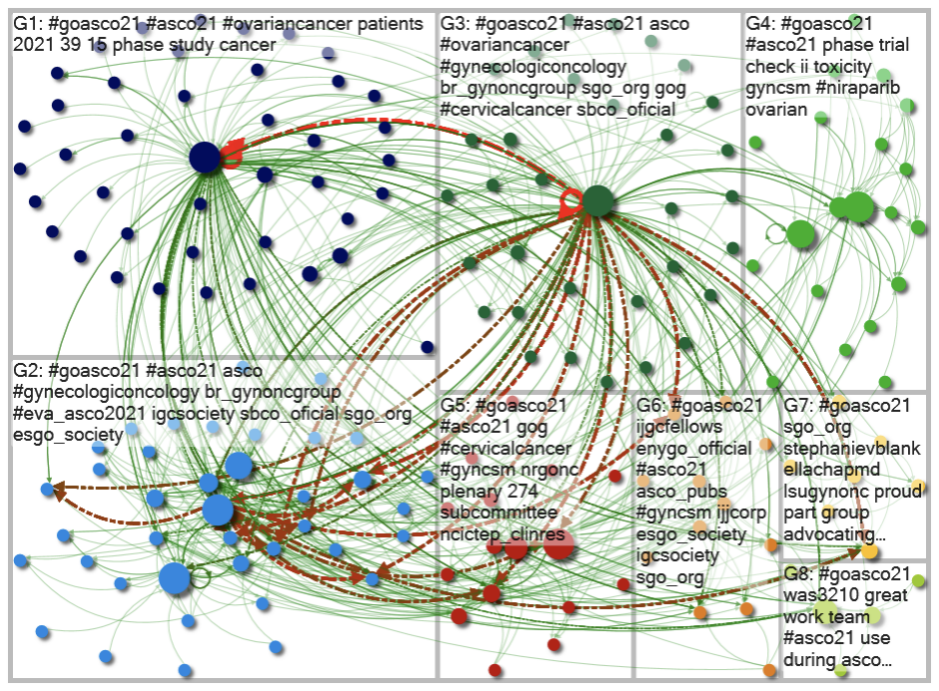
A visual overview of the #goASCO21 Twitter network. ASCO: American Society of Clinical Oncology; G: group.

The most used hashtag was #goASCO21. Different groups of users talked about various topics using the same hashtags. Green lines between groups indicate their relationship and influence on other users. Figure 2 illustrates the various communities of users who shared and tweeted #goASCO21, and all the groups have many green lines between them, indicating that the users were tweeting and mentioning one another. In addition, group 3 strongly influences other groups (red lines), especially group 2. Only 1 circle is more prominent than others in groups 1 and 3, indicating that these users were more influential. Other groups have circles of variable size, showing no clear influential user. In Figure 2, group 3 has more relationships (edges) with other groups than in Figure 1. The most promoted hashtags by group 3 in 2020 were #goASCO20, #ASCO20, #ASCO, and #gyncsm and in 2021 were #goASCO21, #ASCO21, #ASCO, and #ovariancancer.

In 2021, the circles representing Twitter users were spatially arranged in a more balanced way, indicating that there were more users among the different groups in 2021. The increased lines between them illustrate an increase in cross-group discussion.

### Overview of Network Metrics

Table 1 summarizes the network metrics for #goASCO20 and #goASCO21. The 240 tweets using #goASCO20 had 150 unique users and 2519 total edges. The 243 tweets using #goASCO21 had 174 unique users and 2062 total edges. A 16% (174 vs 150 unique users) increase in Twitter use was observed between 2020 and 2021. In 2020, the tweets formed 5 types of edges (mentions, retweets, replies, mention in retweets, and quote tweets) in which #goASCO20 was tagged. These tweets were mentioned 903 times, replied to 75 times, retweeted 367 times, and mentioned in retweets 934 times. In 2021, the tweets also formed 5 types of edges in which #goASCO21 was tagged: these tweets were mentioned 870 times, replied to 360 times, retweeted 33 times, and mentioned in retweets 556 times. To determine if the increase in responses to tweets using the #goASCO21 hashtag compared to the #goASCO20 hashtag was statistically significant, a 2-proportion *z* test was conducted. We compared the proportion of responses for each hashtag (360/2062, 17.5% for #goASCO21 and 75/2519, 3% for #goASCO20). The test resulted in a *z* value of approximately 16.63 and a *P* value <.001, indicating that the difference in response rates is statistically significant.

**Table 1.** Overview of network metrics (#goASCO20 and #goASCO21).

Graph metric	#goASCO20, n	#goASCO21, n	Change, n (%)^a^
Graph types	Directed	Directed	—^b^
Vertices (unique users)	150	174	24 (16)
Unique edges	505	505	0 (0)
Edges with duplicates	2014	1557	−457 (−22.7)
Total edges	2519	2062	−457 (−18)
Edge types	5	5	0 (0)
Mentions	903	870	−33 (−3.6)
Mentions in retweet	934	556	−378 (−40.5)
Replies	75	360	285 (380)
Retweets	367	33	−334 (−91)
Tweets	240	243	3 (1.2)

a The denominator (N) is the #goASCO20 value.

b Not applicable.

Table 2 presents an overview of the top 10 users promoting #goASCO20 and #goASCO21. This study identified 10 influential users based on their location in the network and their “betweenness centrality” score. The rank column orders the users by their “betweenness centrality” score, which reports the influence a user exerts on other users. The “in-degree” value depicts the number of times other users have mentioned an account in their tweets. Users having a high “in-degree” value means that other Twitter users consider them to have high levels of trustworthiness. For example, the user who ranked first in 2020 (@esragbilir) has been mentioned 30 times by other users. The “out-degree” value measures the number of times users mention other users in their tweets. The user who ranked first in 2020 had mentioned other users 90 times in her tweets. The top 3 users in the 2020 ranking (@esragbilir, @Bhandoria, and @ChristinaUwins) belong to the accounts of 3 authors of this study. They have a similar level of trustworthiness, and the first in the 2020 ranking is the user who has mentioned other users the most. The fourth rank in 2020 belongs to @ASCO, the user with the highest level of trustworthiness because of its high “in-degree” value.

**Table 2.** Overview of top users (#goASCO20 and #goASCO21).

Rank	#goASCO20					#goASCO21				
	User	In-degree value	Out-degree value	Betweenness centrality score	Followers, n	User	In-degree value	Out-degree value	Betweenness centrality score	Followers, n
1	@esragbilir^a^	30	90	7688.582	1355	@esragbilir^a^	17	110	13315.076	1355
2	@Bhandoria^a^	30	58	4150.223	1174	@Bhandoria^a^	37	70	11371.626	1174
3	@ChristinaUwins^a^	30	52	2704.416	888	@BatistaTP	10	52	2103.704	727
4	@ASCO	43	1	1686.453	125,888	@DrFMartinelli	14	15	1474.510	709
5	@XXXXX^b^	4	53	1459.960	1329	@gyncsm	14	16	1403.206	5726
6	@GOG	9	5	876.668	824	@AinhoaMada	19	6	1391.266	351
7	@RossFH	18	7	846.868	836	@drmnevsmne2	8	4	1104.949	428
8	@AinhoaMada	7	1	840.000	351	@PayamKashiMD	11	35	1040.534	2523
9	@BatistaTP	12	12	729.352	727	@was3210^a^	11	4	806.254	9943
10	@gyncsm	9	9	703.734	5726	@dsmgyo	12	9	659.031	1246

a Project team members.

b Twitter handle anonymized.

The user who ranked first in 2021 (@esragbilir) was mentioned 17 times by other users in their tweets and mentioned other users 110 times in her tweets. The top 2 users in the ranking in 2021 belong to the accounts of 2 authors of this study, as in the previous year. @Bhandoria had a higher level of trustworthiness than the first user in the ranking, and @esragbilir mentioned other users more than @Bhandoria. The third in rank is @BatistaTP, a gynecologic oncology surgeon. The fourth place in the ranking in 2021 belongs to @DrFMartinelli, a gynecologist specializing in oncology. The fifth place in the ranking belongs to @gyncsm, a community for those impacted by gynecologic cancers.

Table 3 provides an overview of the top 20 cowords used with #goASCO20 and #goASCO21.

**Table 3.** Overview of the top 20 cowords used with hashtags #goASCO20 and #goASCO21.

Rank	#goASCO20			#goASCO21		
	Word 1	Word 2	Count, n	Word 1	Word 2	Count, n
1	#gyncsm	#some4gynonc	130	#asco21	asco	88
2	bhandoria	christinauwins	106	#goasco21	#eva_asco2021	72
3	#womeninstem	#gyncsm	100	#goasco21	#asco21	72
4	christinauwins	ilkerselcukmd	94	sbco_oficial	br_gynoncgroup	60
5	asco	#asco20	92	#eva_asco2021	#sbco	60
6	gynaecological	ncology	89	#sbco	#asco21	60
7	use	#goasco20	88	sgo_org	gog	59
8	#goasco20	#asco20	83	asco	#gynecologiconcology	58
9	during	#asco20	80	gog	esgo_society	56
10	follow	use	79	esgo_society	essonews	56
11	#some4gynonc	#somedocs	69	essonews	sbco_oficial	56
12	#somedocs	#medtwitter	69	br_gynoncgroup	ijgconline	56
13	#asco20	#gyncsm	66	ijgconline	igcsociety	56
14	promote	raiseawareness	63	igcsociety	gyncsm	54
15	raiseawareness	#gynecologiconcology	63	#asco21	#goasco21	42
16	shared	photos	58	#asco21	#gyncsm	31
17	photos	app	58	christinauwins	was3210	25
18	app	photo	58	#cervicalcancer	#endometrialcancer	25
19	esragbilir	bhandoria	55	#cervicalcancer	#goasco21	24
20	#goasco20	promote	55	#goasco21	clin	24

In 2020, the cowords used the most with the studied hashtag were #gyncsm and #some4gynonc (130 times). #Gyncsm is a community for those impacted by gynecologic cancers. #Some4gynonc is a social media group promoting the goal of curing gynecologic cancer globally. In second place, 2 users were mentioned 106 times with #goASCO20: @Bhandoria, a gyneoncologist and obstetrician, and @ChristinaUwins, a surgeon and senior research fellow in robotic gynecologic oncology. In third place, 2 hashtags (#womeninstem and #gynscm) were used 100 times. The hashtag #womeninstem promotes women and gender equality in science, technology, engineering, and mathematics. In fourth place, there were 2 users, both of whom were mentioned 94 times with #goASCO20. Finally, the fifth place belongs to the hashtags #asco and #goasco20, and both were mentioned 92 times with #goASCO20.

In 2021, the cowords used the most with #goASCO21 were #asco21 and #asco (88 times). In second place, #asco21 and #eva_asco2021 were used 72 times. The first refers to the ASCO, and the second (#eva_asco2021) refers to a group focused on gynecologic tumors from Brazil. The third most used cowords (72 times) were hashtags that promoted the spread of clinical knowledge (#goASCO21 and #asco21). The fourth-ranked cowords (60 times) were sbco_oficial, a Brazilian society of oncologic surgery, and br_gynoncgroup, a Brazilian gynecologic oncology group. The last word pairs in the top 5 most used cowords were #eva_asco2021, a group focused on gynecologic tumors from Brazil, and #sbco, a hashtag used to refer to the Brazilian Society of Oncologic Surgery.

## Discussion

### Principal Findings

This study hypothesized that introducing a new hashtag specific to gynecologic oncology could provide a focus for tweeting about gynecologic cancers. A new hashtag, #goASCO20, was presented on Twitter during the ASCO 2020 virtual conference and was replaced with #goASCO21 in 2021. Conference attendees were encouraged to use these new hashtags when discussing anything related to gynecologic cancers. The use of these new hashtags was actively encouraged. Users who promoted the hashtag in 2020 did not tend to respond to tweets but, in 2021, increased their response rate (75 times in 2020 vs 360 times in 2021). This shows that the gynecologic oncology community started engaging better in the second virtual congress. Consistent use of hashtags has enhanced Twitter engagement, as evident in the study by Morgan et al [15]. The cumulative number of impressions for #ASCO16 was 468.2 million compared with approximately 1.12 billion for #ASCO20 [15]. We predict a similar growth of #goASCO if its use is continued.

COVID-19 played a crucial role in social media use among the oncology community. It forced the annual meeting to go entirely virtual. As evidenced by our study, the conference attendees used social media channels more to interact.

The 2 users who promoted the hashtags the most were the same in 2020 and 2021. It should be noted that @esragbilir and @Bhandoria significantly increased their “betweenness centrality” score, indicating that their location in the network became more influential among the users. Establishing a core social media team that actively promotes it is essential.

### Strengths and Weaknesses

This is the first study where a new hashtag was introduced and social media interaction was measured. This study contributes to the literature on this topic, highlighting how networks can be used to spread trustworthy information and share relevant information among the scientific community on Twitter.

A limitation of this study is that it was not designed to assess the validity of any tweets but to evaluate the success of promoting the use of a gynecologic oncology–specific hashtag in increasing interaction between individual Twitter users and organizations. Misinformation on Twitter is a recognized phenomenon; future studies should investigate whether the quality and quantity of discussion are affected [16]. Since the inception of oncology hashtags, we acknowledge the existence of the gynecology-specific hashtag #gyncsm [17]. We created the #goASCO hashtags to study its impact as #gyncsm is used more by patients with gynecologic cancer and their advocates [18]. We should have examined the effect of #gyncsm during these virtual meetings, and this may be seen as a weakness, with no comparator group being available. Lastly, some of the “influential Twitter users” named in the results included a few authors. However, this is not aimed at self-promotion but is part of the results’ description.

### Conclusion

The use of a gynecologic cancer–specific hashtag helped facilitate discussion on topics in gynecologic oncology on Twitter during the 2020 and 2021 ASCO virtual meetings. This impact was visible in the social network analysis.

## Supplementary material

10.2196/45291 Multimedia Appendix 1 Terms related to social media research.

## Abbreviations

ASCO: American Society of Clinical Oncology
ESGO: European Society of Gynaecological Oncology
