# Supplementary material for: Impact of a New Gynecologic Oncology Hashtag During Virtual-Only ASCO Annual Meetings: An X (Twitter) Social Network Analysis
Source: JMIR Med Educ. 2024 Aug 14;10:e45291. doi: 10.2196/45291 (PMC11339558; doi:10.2196/45291)
Supplement: Multimedia Appendix 1 [file mededu-v10-e45291-s001.docx]

| **Metric** | **Definition** |
| --- | --- |
| Directed and undirected Graph Type | A directed X (Twitter) network is where the relationship has a direction, e.g., if User 1 retweets User 2, there is a link from User 1 to User 2 |
| Vertices | In a X (Twitter) network, a vertice is the individual X (Twitter) user, also called a 'node.' |
| Unique Edges | Edges indicate relationships and unique edges count these relationships between users only once. For instance, if User A mentions User B several times, it will only be counted once. |
| Edges With Duplicates | Edges with duplicates will include all relationships, including repeat mentions, retweets, and replies. |
| Total Edges | The total edges column will show user relationships, e.g., mentions, retweets, and replies in a given network. |
| Number of Edge Types | There can be multiple types of edges, such as mentions, retweets, and replies, and the types of edges can be counted. |
| Mentions | X (Twitter) users can '@' mention one another, and the mentions count shows the total number of mentions in a network. |
| Replies to | Users can reply directly to other users' tweets, which can be measured and counted. |
| Retweet | A retweet is when another user's tweet is reshared, and the total retweets can be counted in a given network. |
| Tweet | A tweet is a unique message poster by a user that does not contain an ‘@’ mention. |
| Mentions-In-Retweet | A particular type of retweet allows users to mention other users in tweets, which can also be counted. |

Appendix 1 - Terms related to social media research
